# The Observer. No. II

**Published:** 1817-06

**Authors:** 


### JOURNAL AND REVIEW.

VOL. III.] JUNE, 1817. [NO. 18.

### PART I.

### ORIGINAL COMMUNICATIONS.

### THE OBSERVER. No. II.

"Periculosum est credere et non credere: Ergo exploranda est veritas multum priusquam stulta pravé judicet sententia." PHÆDRUS.

### ART. 4. CARDIAC DISEASES.

IT would seem passing strange, that the derangements of so important an organ as the heart should have been so long overlooked, that their investigation is yet in its infancy! Attention being now roused, however, to this point, a day scarcely passes without some interesting fact being proclaimed, some phænomenon unravelled, that lets in an additional ray of light on the obscurity in which the subject has been hitherto involved. The anomalous, and indeed unaccountable symptoms which so frequently attend structural lesions of the circulating organ, have doubtless proved *ignes fatui* that led the medical attendant not only far astray from the real source of these phænomena, but into snares that too often proved fatal to the patient. The day is probably not far distant, when nine-tenths of those diseases denominated "nervous" shall be placed to the account of derangements in the balance of the circulation, and functional or organic lesions of the heart; and consequently, when a complete revolution shall take place in the past and present modes of treatment. It was but last year that, in the "intellectual city" of Edinburgh, a man was treated with bark, valerian, opium, assafœtida, wine, and æther, till the very hour of his death; the last dietetic prescription of the physician being in these words: "*Let him have a bit of steak*;" after which immediately follows—"Died at seven o'clock."*

The pericardium contained from six to eight ounces of muddy serum, with flakes of a curdy matter. The heart was greatly altered in texture. The whole parietes of the right auricle, and the anterior side of the corresponding ventricle, were found converted into a greyish-coloured substance, in consistence like the prostate gland. No vestige of the natural tunics was here to be found. Similar changes, of more or less extent, were found in the other side of the heart, while the abdomen presented a mass of diseased mesenteric glands and disorganized intestines. Four days before his death, so perfectly had the cardiac disease simulated hypochondriasis, that Dr. Duncan, an eminent, enlightened, and skilful physician, entertained not the slightest suspicion of the mask, and actually tried Kæmpf's method of treating hypochondriac affections. Even when death took place, "my idea (says Dr. D.) of his present complaints was, that effusion had taken place in the brain, or upon the spinal marrow, oppressing the powers of life." This great example of deception, in the very cradle of medical science, may afford some faint idea of the victims which have fallen, and daily fall, at the shrine of this Protean class of maladies! The case deserves yet another remark. Dr. Duncan says, that there was *no symptom* of cardiac affection during life. Whether the thorax and abdomen were examined by percussion and compression is not stated; but surely a pulse ranging *constantly* from 104 to 108; a tongue covered with a brownish crust; a constipated state of bowels; a gnawing pain in the region of the stomach, aggravated on taking food; great debility; interrupted sleep; want of appetite, &c. all attributed to "being exposed to cold and hard work," were phænomena that might fairly excite the suspicion of something more than met the eye—of something beyond mere hypochondriasis. When to these were added (as on the 19th), head-ach, general weakness and pains, "*pain even on slight pressure a little below the scrobiculus cordis and*

* Edinb. Med. Surg. Journal, No. 45, p. 46.

*to the left side,*" the proofs of *organic* derangement going on internally were accumulating. When four days after this, " the pulsation of his heart was distinctly felt, even when lying towards the right side," with the pulse 107, there certainly would appear to us sufficient grounds for laying aside the idea of hypochondriasis.

The next remarkable example of cardiac deception is related by Dr. Young, in the first number of the *Journal of Science and the Arts.* — J. O. ætat. 49, sanguine temperament, was admitted into the Edinburgh Infirmary in Dec. 1815, labouring under *eczema mercuriale,* that had supervened on the application of ung. hyd. nit. to the eyelids, and to an ulcer on the leg. *Pulse frequent, intermittent, and variable;* heat natural; great prostratio virium; and imperfect sense of touch. These symptoms were attributed to *erethismus* from mercury. Antimonials, opium, sarsa, cinchona, mineral acids, nutritive diet, open air. By these means the cutaneous affection was removed; but the irregularity of pulse and prostratio virium continued. Three weeks after admission, he experienced a febrile attack, with cough, dyspnœa, universal pains, enlargement and tenderness in the right hypochondriac and epigastric regions, which was considered hepatic derangement. The febrile state was removed by mild and common means; but he expired suddenly on the 23d of January.

*Post mortem.* Sixteen ounces of a reddish fluid in the right cavity of the pleura; recent marks of inflammation in the right lung. In pericardio, ten ounces of a turbid and very red fluid. Indications of recent inflammation on the serous membrane of the heart, where it covers the appendix of the right auricle; the opposite portion of pericardium in a morbid state. The heart double its natural size, weighing 28 ounces. Foramen ovale so open that the auricles formed one extensive bag. The cavæ and pulmonary veins were enlarged. Tricuspid valve ossified in some parts. Pulmonary artery much larger than the aorta; its semilunar valves completely ossified. Cavity of aortic ventricle of a natural size, but its parietes thickened. Aorta natural. Sixteen ounces of a straw-coloured fluid were found in the abdomen. Liver pale, but otherwise healthy; other abdominal viscera healthy. The only particulars of this man's previous history that could be collected were, that during the last eighteen years he had had several attacks of thoracic inflammation; that four years ago he experienced a slight apoplectic attack, which left him less vigorous, with a sensation of numbness over the whole body. He never had a livid complexion, or other symptom of cardiac disease than the peculiar state of the pulse.

These two cases are sufficient to excite the attention of the Faculty in general to the investigation of cardiac affections. In every case of doubt, and God knows these are but too common, the thorax and abdomen should be examined with the utmost attention in every position. The trouble will be amply rewarded by the insight which will be gained; and many an affection of an internal organ that, from verbal description, would remain veiled from the view, will be thus developed.

### Art. 5. Carpo-Pedal Spasm, *from Dentition.*

This curious affection has been very imperfectly hinted at by Dr. Underwood, who describes it as merely a "swelling of the tops of the feet and hands," of little importance, and going off on the appearance of the teeth. Dr. Kellie, of Leith, in the 12th vol. of the Edinburgh Journal, gives a much more particular account of the disease, as accompanied frequently by "a spastic contraction of the flexor muscles of the thumbs in the upper, and of the toes in the lower extremities." Dr. Clarke, in his Commentaries on the Diseases of Children, also takes notice of the complaint, as a *peculiar species of convulsion* little known to practitioners, though occasionally fatal in its consequences.

The Observer has lately seen an exquisitely marked case of this disease, for which he can give no better name than that prefixed to the article.

The patient was a delicate female child, nineteen months old, and cutting three or four teeth at the time. She had been ill eight days before the Reporter saw her; but according to the mother's account, there was little or no variation in the symptoms all the time. When first seen, the child three or four times in the hour was seized with spasmodic affections of the respiratory muscles, consisting of repeated attempts to fill the chest, during which she threw herself back, as in opisthotonos, and appeared as though she would be suffocated. These fits would last ten or twelve minutes, after which the child was somewhat easier, but always fretful and peevish. The backs of the hands and insteps were swollen and hard. The thumbs were rigidly contracted, and locked across the palms of the hands. The toes were bent down towards the soles of the feet, and both wrists and ankles were rigidly bent by the flexor muscles, and kept permanently so. The little patient could take no food: she was slightly feverish; the bowels torpid; and the stools clayey, slimy, and offensive. The eyes looked very heavy and inanimate. The child extremely irritable and restless both by day and by night.

For reasons unnecessary to mention, no very active means were employed for some days, during which the child got worse, and was evidently declining fast. Hydrencephalus or eclampsia being now dreaded, leeches were applied to the temples; calomel and antimonial powder were exhibited thrice a day, and the warm bath was employed in the evening. After the bath and bleeding, the spasms went off for twelve hours entirely, and the child took some food. They returned, however, but not so violently as before, and the convulsive respiration was much relieved. The calomel and antimony were continued, as was the warm bath; and the gums were deeply scarified wherever a tooth appeared to be coming. From this time the child gradually recovered, and on the 18th day the spasms had entirely disappeared.

### Art. 6. Hæmorrhoids.

Much as has been written respecting Hæmorrhoids, their nature is by no means well understood. That the discharge from the hæmorrhoidal vessels is often salutary, cannot be doubted; but that a more convenient drain from the system might be found in some *vicarious* evacuation, will hardly be questioned. From an anatomical survey of the seat of these swellings, it is more than probable that their causes partake a good deal of a mechanical nature; and whether or not this be the case, I can confidently assert, that their prevention and cure will be more frequently effected by a simple mechanical mean, than by all the medicines in the Pharmacopœia. This is *pressure* steadily and long, but very easily applied.—A gentleman who for years was harassed by hæmorrhoidal projections and discharges, has got entirely free from both, by a perseverance in the above plan. It is simply this:—A kerchief (No. 1), is to be bound moderately tight round the loins, while the end of another (No. 2), is to be made secure to the former, where it crosses the lumbar region. The hæmorrhoidal tumour is then to be pressed up, if possible, within the sphincter, while the person lies in the horizontal position. If the hæmorrhoids cannot be entirely returned, which is too often the case at first, then a pledget of lint spread with the cerat. sup. plumbi, well saturated with liq. plumbi acet.; and if there be much pain, with powdered opium, is to be applied to them, and the kerchief (No. 2), to be drawn as tightly over them as the person can bear, making the remaining end of it fast to the kerchief (No. 1), by taking two or three turns above one groin. There will thus be a steady, elastic pressure made all along from the coccyx to the pubis, and such a support will be given to the parts, that a person who could not previously walk without great pain, will now move about with ease. This apparatus is to be worn constantly as a *preventive*; for it is hardly possible for any hæmorrhoidal tumours to protrude while it is applied.

That some other evacuation should be substituted for the suppressed hæmorrhoidal discharge, I am aware; and it appears that occasional abstinence and open bowels are all that is necessary. Five grains of the mercurial pill every second or third night, for a week or ten days at a time, will keep up a healthy secretion in the track of the intestines, and a salutary influence on the hepatic system, where want of energy seems often the cause of turgescence in the hypogastric vessels.

The above plan has been tried in three other instances with complete success.

### Art. 7. Observations on Mercury.

Various have been the disputes respecting the operation of mercury on the human system. A *stimulant* property has been pretty universally attributed to this mineral, apparently from its quickening the vascular action, and "exciting an artificial fever."* Hence, says the "Enquirer" [*loco citato*] "its efficacy in remittent and continued fevers is very equivocal. At the commencement of those diseases I believe that it does mischief, if exhibited in any form to exert its power on the salivary glands *alone*." In the first place, I would be glad to know the Enquirer's authority for this assertion, unless he means Dr. Saunders, whose prejudices are only equalled by his inexperience in tropical fevers, where the remedy is employed. The great majority of tropical writers are in favour of mercurial influence on the system in fevers; and those who are adverse to this measure, do not accuse mercury of any positive mischief, but only say that it is inefficacious, except as an evacuant. But I would ask the En-

* Edinburgh Med. and Surg. Journal, vol. vi, p. 187. "The Enquirer, xviij."

QUIRER, if he ever saw mercury, in any form, affect "the salivary glands *alone?*" And this leads me to the immediate object of this article; namely, to observe that mercury is never more an evacuant than when it produces ptyalism. Narrow, indeed, is that view of the mercurial action, which stops short at its quickening the pulse and "exciting an artificial fever." The fact is, that ptyalism is merely a symptom that the salivary glands are affected, in common with every other gland, and every secreting and excreting vessel in the system. Thus flood-gates are opened in all directions, and every part of the human fabric experiences a rapid diminution. This effect is still farther increased by the ptyalism preventing any supply of solid nutriment which the patient or friends might wish to introduce. Here then is the *ratio agendi* of a medicine which is branded as a stimulant, and even as a *febrific;* while some of the most acute and able physicians of the age have acknowledged its decided *febrifuge* qualities. Among a host of recent authorities, I may allude to the inestimable work of Dr. Armstrong on Typhus, &c. where the efficacy of mercury is developed from wide experience. If Dr. Dickson pronounce it *inefficacious* in certain forms of tropical fever, [he has not ventured to accuse it of injurious effects] Dr. Denmark, another physician to the fleet, of equal rank and authority, speaks differently.—"Viewed in any way, the utility of mercury is incontrovertible. Whether it produce catharsis, when exhibited with a view to salivate, or salivate when intended to act as a cathartic, the result, in either case, will be beneficial." *Med. Chir. Trans.* vol. vi. p. 306.

Nothing can expose the absurdity of the stimulant hypothesis more conspicuously than the effects of mercury in dysentery. Here we have a long track of intestines in a state of high irritation, if not inflammation, with symptomatic fever, &c ; and what do we in such a case? Why, apply calomel to the very parts that are irritated and inflamed! Yes, we apply that stimulant, febrific mercury, not only as a purgative, but as a sialagogue, in a disease where there are local inflammation and general fever;—and, *mirabile dictu!* we remove both, as soon as ptyalism is established!

A Case

